# Assessing the Air Pollution Mitigation Potential of Urban Trees in Ghana’s Central Region

**DOI:** 10.1155/tswj/9655268

**Published:** 2025-12-28

**Authors:** Francis Kwaku Nkansah, Ebenezer J. D. Belford, Jonathan Nartey Hogarh

**Affiliations:** ^1^ Department of Environmental Science, University of Education, Winneba, Ghana, uew.edu.gh; ^2^ Department of Theoretical and Applied Biology, Kwame Nkrumah University of Science and Technology, Kumasi, Ghana, knust.edu.gh; ^3^ Department of Environmental Science, Kwame Nkrumah University of Science and Technology, Kumasi, Ghana, knust.edu.gh

**Keywords:** air pollution tolerance index, anticipated performance index, biochemical responses, bioindicators, roadside trees, Winneba

## Abstract

Air pollution is a major environmental concern in urban areas, necessitating the identification of tree species capable of mitigating its effects. This study assessed the Air Pollution Tolerance Index (APTI) and Anticipated Performance Index (API) of four common urban tree species (*Albizia lebbeck* (L.) Benth., *Azadirachta indica* A. Juss, *Khaya senegalensis* (Desr.) A. Juss, and *Senna siamea* (Lam.) H.S. Irwin & Barneby) to evaluate their potential for air pollution control. Leaf extract pH was determined by homogenizing 5 g of leaf tissue in 10 mL deionized water and measuring with a calibrated digital pH meter. Relative leaf water content (RWC) was calculated using fresh, turgid, and dry weights of leaf samples. The ascorbic acid (AA), total chlorophyll (TC), and carotenoid contents were determined spectrophotometrically using 721 Vis spectrophotometer. API was determined by integrating APTI with morphological and socioeconomic traits. One‐way ANOVA and correlation matrices were employed to analyze variations across roads and the relationships among parameters, respectively. The findings revealed significant variation in biochemical responses among the studied species. Principal component analysis (PCA) identified AA, RWC, and TC as consistent key drivers of variation across all roads, while leaf pH exhibited site‐specific influence. The mean APTI values of the four tree species varied from 5.39 to 8.96. Based on the APTI, the tree species were classified as either tolerant (> 7.5), intermediate (6.5–7.5), or sensitive (APTI < 6.5). *A. indica* exhibited consistent RWC and chlorophyll levels, earning the highest API score (81.25%) and an “Excellent” performance rating, making it a potential candidate for urban greenbelt development. *K. senegalensis* can be used as sentinel species in urban greening because of its general sensitivity to the pollutants. *A. lebbeck* and *S. siamea* showed strong potential as bioindicators because of their physiological responses to pollution stress. This study introduced a new classification range for using roadside trees as bioindicators of vehicular pollution, particularly under conditions of low APTI.

## 1. Introduction

Air pollution has become a critical global environmental and public health concern, intensified by rapid urbanization and increased dependence on vehicular transportation. In many developing countries, insufficient public transport systems have driven a rise in private vehicle use, significantly degrading air quality [1, 2]. Vehicular emissions are now among the leading contributors to air pollution globally, surpassing many other human activities [3]. These emissions release harmful pollutants such as sulfur dioxide (SO₂), nitrogen oxides (NO_x_), carbon monoxide (CO), carbon dioxide (CO₂), volatile organic compounds (VOCs), smoke, dust, fumes, and aerosols, all of which adversely impact human health, plant physiology, and ecosystem stability [4].

A growing body of research has linked air pollution to serious health outcomes in both humans and plants [5–7]. Exposure to these pollutants has been associated with respiratory and cardiovascular diseases, compromised immune systems, reproductive challenges, and elevated mortality rates [6, 7]. Notably, maternal exposure to particulate matter (PM₂.₅ and PM₁₀), CO, and SO₂ during pregnancy has been correlated with reduced newborn telomere length, a marker of long‐term health risks [8].

Trees growing along roadsides serve as buffers against vehicular emissions. However, prolonged exposure to pollutants can impair plant physiological functions such as photosynthesis, transpiration, stomatal conductance, and DNA integrity, while also leading to the accumulation of heavy metals in plant tissues [9–13]. Pollutants have also been shown to reduce chlorophyll, carotenoid, proline, and protein content in leaves. Ascorbic acid (AA), a key biochemical marker of plant stress, has been closely linked to pollution levels, emphasizing its relevance in environmental stress assessments [14, 15].

Although conventional methods such as fixed monitoring stations provide accurate data, they are costly and require specialized expertise. Satellite‐based remote sensing offers broad coverage but lacks the spatial resolution needed to detect street‐level pollution. Similarly, models like i‐Tree Eco estimate ecosystem services and pollutant removal but may oversimplify site‐specific ecological interactions [16]. In contrast, urban trees present a cost‐effective and environmentally friendly alternative for monitoring air quality. Through their leaves, bark, and canopy structure, trees act as passive biofilters, absorbing gases and trapping particulate matter (PM) [17–19].

Biochemical indices such as the Air Pollution Tolerance Index (APTI) and Anticipated Performance Index (API), derived from leaf physiological parameters, have been validated in studies conducted in Tehran and other urban areas [20]. For example, species like *Morus alba* and *Salix babylonica* were classified as tolerant or sensitive based on their physiological responses to pollution [20]. This approach is especially suitable for resource‐limited urban areas, as it captures localized pollution patterns with high spatial resolution and requires only basic laboratory facilities.

Urban trees play a key role in mitigating air pollution by intercepting airborne pollutants, absorbing gaseous emissions, and accumulating PM on leaf surfaces [18, 19]. However, their effectiveness varies depending on species‐specific physiological, biochemical, and morphological characteristics [9, 21]. Despite continuous exposure to vehicular emissions, roadside plants function as natural air purifiers and are valuable assets in urban greening initiatives [22, 23].

To assess the pollution mitigation capacity of tree species, researchers employ the APTI, which evaluates AA content, chlorophyll concentration, leaf extract pH, and relative water content (RWC) [24, 25]. This index categorizes plants as tolerant, intermediate, or sensitive to air pollution, helping identify species suitable to be used as bioindicator and for pollution mitigation. Complementing the APTI, the API incorporates additional morphological and socioeconomic factors to rank species’ performance as poor, very poor, good, very good, excellent, or best [26–28].

Although several studies have applied APTI and API in different regions including those by [29] on the morphological responses of urban trees to traffic in Karachi, Pakistan; [30] on roadside trees in Nigeria; and [11] on the biochemical impacts of vehicular emissions in Pakistan. Bala et al. [24] also used API models for tree selection in India. However, in Ghana, particularly within the Effutu Municipality, studies have largely concentrated on ambient air quality assessments (e.g., PM, gaseous pollutants) [31, 32] without systematically linking them to vegetation responses.

Winneba was selected for this study because of its location along key highways experiencing increasing vehicular activity, which contributes significantly to local air pollution [31]. Despite this, research on using urban trees for pollution mitigation in this region remains limited. Understanding the role of local and widely distributed tree species in air quality management can inform urban planning and greenbelt development.

Therefore, in this study we assessed the APTI and API of *Albizia lebbeck* (Lebbeck Tree), *Azadirachta indica* (Neem tree), *Khaya senegalensis* (African Mahogany), and *Senna siamea* (Siamese Cassia) along three major highways in Winneba and a control site at the University of Education, Winneba. These species were selected because of their commonest and abundance [33] in the study area. While this selection provides a representative baseline for assessing air pollution tolerance in commonly encountered roadside trees, it inevitably limits broader generalization.

This research was guided by the following key questions: What are the species‐level variations in physiological and biochemical parameters? How do APTI and API vary among the selected tree species? It was hypothesized that tree species with higher APTI and API will demonstrate greater potential for air pollution mitigation and can serve as indicator.

The objectives of this study were to determine species‐specific variations in biochemical parameters in response to air pollution and estimate the APTI and API of selected urban tree species. This study presents improved classification ranges for selecting roadside tree species in urban greening projects for the first time, especially in situations with low APTI.

## 2. Materials and Methods

### 2.1. Study Area

**Figure 1:**
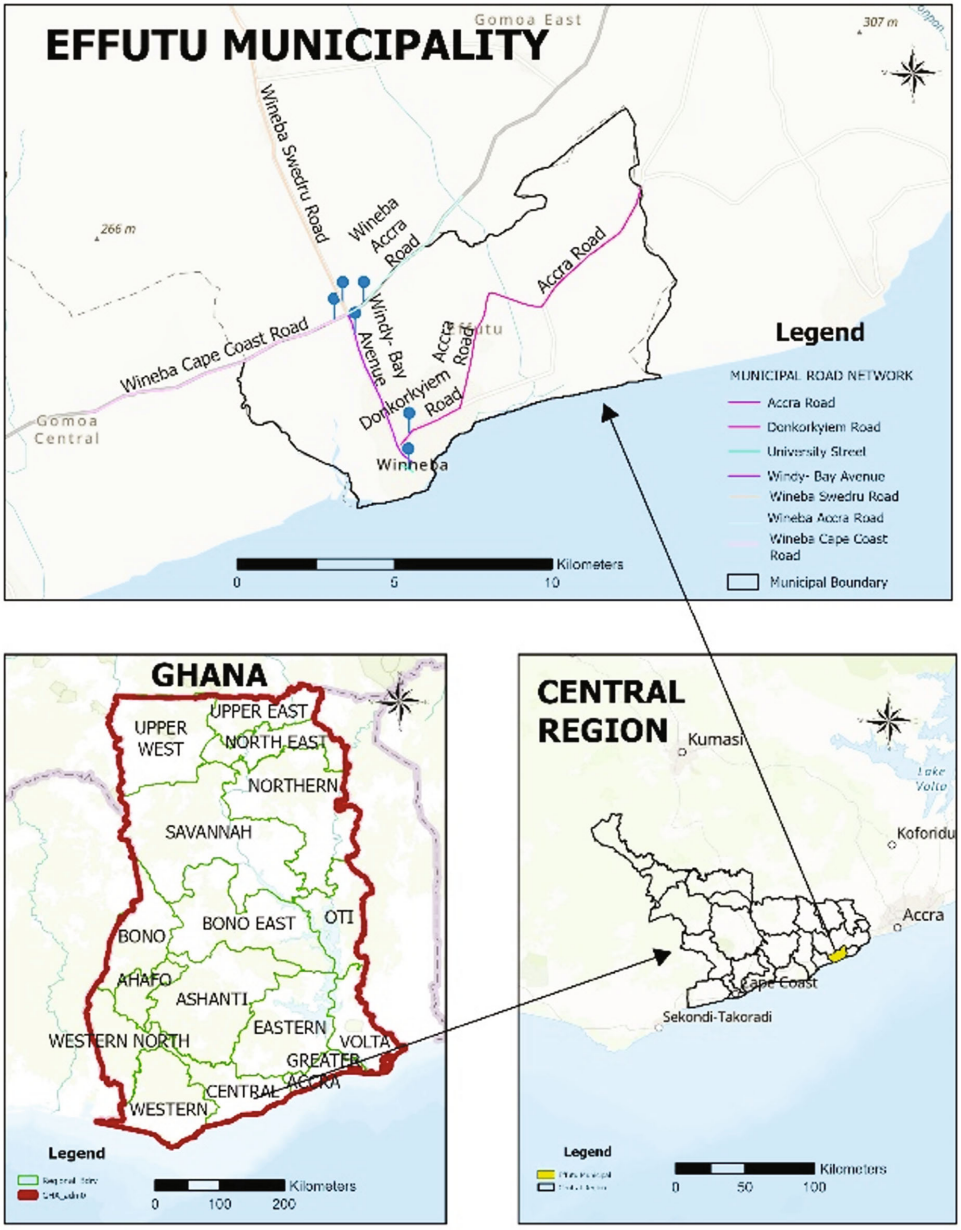
Map of study area indicating the highways [33].

Winneba is located in the Central Region of Ghana and is a coastal town bordering the Gulf of Guinea to the south and surrounded by rolling hills to the north (Figure 1) [33]. The city lies between 5°20 ^′^ and 5°35 ^′^ north latitudes and 0°37 ^′^ and 0°48 ^′^ west longitudes. The city covers an area of about 17.5 km^2^ and has about 60,000 inhabitants [34]. Winneba has a tropical climate characterized by warm and humid conditions, with average annual temperatures between 24°C and 30°C and average annual rainfall of about 850 mm. Winneba is close to major highways and is a center for vehicular traffic, which is known to contribute to local air pollution, especially along major roads [31]. Description of the various highways in Winneba during the study period is presented in Table 1.

**Table 1:** Description of sampling highways in Winneba during the study period.

**Sampling site (arterial roads)**	**Road distance (km)**	**Average total traffic count per lane per hour**	**Latitude (N)**	**Longitude (W)**	**Congestion classification**
Highway I	4.1	1,388	05°22.649E ^′^	000°38.432 ^′^	Heavy
Highway II	3.2	784	05°23.178 ^′^	000°38.645 ^′^	Medium
Highway III	10.2	604	05°20.929 ^′^	000°37.505 ^′^	Light
Control	1.0	N/A	05°20.316 ^′^	000°37.520 ^′^	Control site

*Note:* Source: [35].

### 2.2. Experimental Design

**Figure 2:**
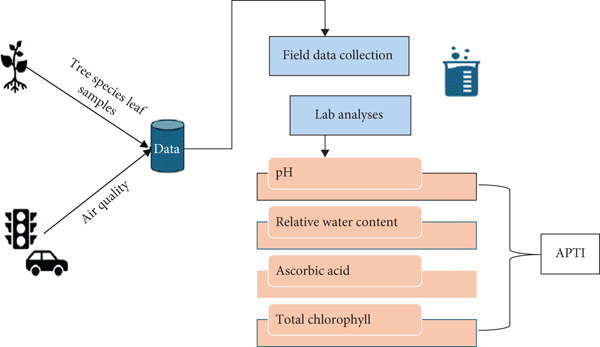
Approach adopted to determine APTI from field data and lab tests.

The following framework (Figure 2) was used to carry out this research: (i) The first step was to collect leaf samples from selected tree species at sites with varying levels of vehicular emissions. Field data on air quality were also recorded to characterize pollution exposure. (ii) A leaf extract pH test was carried out to determine the acidity or alkalinity of the leaves. (iii) The RWC of the leaves was measured to assess the internal water balance and physiological status of the tree species. (iv) An AA test was performed using a spectrophotometric method to quantify the antioxidant capacity of the leaves. (v) Finally, the total chlorophyll (TC) content was determined spectrophotometrically by measuring absorbance at two wavelengths to calculate the concentrations of the TC. The values obtained from these parameters were integrated to compute the APTI, which was then used to classify tree species as tolerant, intermediate, or sensitive to air pollution.

### 2.3. Tree Species and Collection of Leaf Samples

The taxonomical identification of the studied tree species was authenticated by qualified taxonomists, and voucher specimens were deposited at the Kwame Nkrumah University of Science and Technology. Details of voucher numbers and authentication were as follows: *A. lebbeck* (AL‐SW‐001‐2024, AL‐WBA‐002‐2024, AL‐DK‐003‐2024), *A. indica* (AI‐SW‐001‐2024, AI‐WBA‐002‐2024, AI‐DK‐003‐2024), *K. senegalensis* (KS‐SW‐001‐2024, KS‐SW‐002‐2024, KS‐DK‐003‐2024), and *S. siamea* (SS‐SW‐001‐2024, SS‐WBA‐002‐2024, SS‐DK‐003‐2024) [35]. *A. lebbeck*, *A. indica*, *K. senegalensis*, and *S. siamea* were the most common and abundant trees species along the highways in Winneba [35].

*A. lebbeck*, *A. indica*, *K. senegalensis*, and *S. siamea* were the most common and abundant trees species along the highways in Winneba [33]. Leaf samples for physio‐biochemical analysis were collected along the Winneba junction—WindBay Avenue (Highway I), Winneba junction‐Swedru (Highway II), Winneba Central‐Donkorkyiem roads (Highway III), and the University of Education, Winneba South campus served as the control road. *K. senegalensis* was not present at Highway III and as a result, no data were collected. The notation “N/P” has been used to stand for “Not Present”. This activity spanned between May and July 2024 (i.e., major rainy season) before annual shedding plant leaves. For each tree species at both the experimental and control roads, three independent leaf samples were collected and analyzed. All statistical values are presented as mean ± standard deviation (SD) based on three replicates per species per site. Each tree species with diameter at breast height (DBH) of more than 10 cm and height between 5 and 20 m were sampled within a distance of 20 m away from the edge of the roads. Sampling intervals between each tree species replicate ranged from 0.01 to 1 km along each road. Twenty to 30 physiologically active leaves at the third from the tip of the apical bud were picked from the branch of the tree facing the road between 7:00 and 09:00 h at each sampling road. Samples were kept at −40°C until needed.

### 2.4. Ambient Air Quality

The measurement of PM₂.₅, PM₁₀, CO, and NO_x_ concentrations followed the approach described in [31, 35]. Briefly, pollutants were monitored over three daily intervals using EPAM‐7500 and Aeroqual 500 Series monitors, with equipment positioned approximately 1 m above the ground to capture vehicle emissions. Measurements were conducted 5 days per week over a 3‐month period, with data collection rotated among selected streets and averaged over 2‐h periods.

### 2.5. Determination of Physiological and Biochemical Traits

#### 2.5.1. Quantification of Leaf RWC

The method outlined by [36] was used to calculate the total RWC. To measure the fresh weight, leaf samples from each replication of the tree species were weighed. The leaves were submerged in 150 mL deionized water [36] for 18.0 h overnight. To find the turgid weight, they were taken out, dried with blotting paper, and weighed. After that, they were oven (Genlab MINIS/30) dried for 1.0 h at 70°C (a consistent temperature was verified using a thermometer) until they had a constant dry weight. Mettler Toledo PB4002‐S, a Swiss chemical balance, was used to measure the fresh, turgid, and dry weights. The relative leaf water content was calculated using (Equation 1) the formula of [36–38]:

(1) Relative water content,RWC=Fw−DwTw−Dw×100%

Where: FW = fresh weight, DW = dry weight, and Tw = turgid weight.

#### 2.5.2. Determining Leaf‐Extract Acidity/Alkalinity

Leaf samples of 0.5 g was crushed and mashed into a paste using a mortar and pestle. Ten milliliters (10 mL) of deionized (DI) water was used to homogenize it. After filtering the resulting suspension, the pH was determined with a calibrated Jenway 3020 digital pH meter [39, 40].

#### 2.5.3. Determination of AA Concentrations

The AA concentration (mg/g) of the leaves was estimated using 721 Vis spectrophotometer [41, 42]. Fresh foliage was weighed (1 g) and grinded. Extraction solution of 1 g oxalic acid and 0.15 g NaEDTA in 200 mL was prepared. To the ground leaf in a test tube, 4 mL of the extraction solution was added and centrifuge for 15 min at 6000 rpm. The supernatant (1 mL) and 5 mL of 2,6‐dichlorophenolindophenol was mixed with constant shaking and the optical density (OD) of the pink solution (Ps) was measured at 520 nm wavelength. A calibration curve was prepared by using 1% aqueous AA solution, which was diluted to obtain varying concentrations.

#### 2.5.4. Quantification of Chlorophyll and Carotenoid Pigments

The TC contents were determined using the spectrophotometric method [43]. Leaf samples weighing 0.5 g of each tree species replicate were homogenized in 5 mL of 80% acetone solution using a mortar and pestle. The homogenate was then transferred to a test tube and centrifuged at 4000 rpm for 2 min using Chalice Centrifuge by Wagtech International. The resulting supernatant was transferred into a 5 mL volumetric flask and made up to 5 mL using 80% acetone. The color intensity of the green pigment was read using a 721 Vis Spectrophotometer (7052208009, made in England) at wavelengths of 645 nm, and 663 nm against a blank. The TC was calculated as follows [25, 44]:

Total chlorophyll TC=20.28.02 A 645+A 663×V1000×W 

Where A = absorbance of the extract, V = total volume of extract (mL), and W = weight of fresh leaf (g).

### 2.6. APTI of the Tree Species to Vehicular Emissions

The APTI is a measure of a plant species’ inherent ability to withstand and respond to air pollution stress, serving as an indicator of the impacts of air pollutants, particularly in industrial and nonindustrial areas [26, 45]. The APTI is well documented as the primary metric that unravels the effects pollutants on the biochemical properties of plant species indicating their tolerance or susceptibility to air pollution [25, 45, 46].

The leaf extract pH, RWC (%), TC (in mg/g), and AA content (in mg/g) [47] were used to assess the APTI of plant species (tolerance or sensitivity to pollution including vehicular emissions). The calculation of APTI is performed using the expression [39, 40]:

APTI=AATC+pH+RWC10

The classification of APTI into three grades—tolerant (T), intermediate (IM), and sensitive (S)—was based on thresholds derived from the mean and standard deviation (SD) of APTI values, as per [36]. Specifically:

1. Tolerant: APTI > mean APTI + SD (i.e., > 7.5);
2. Intermediate: mean APTI − SD < APTI < mean APTI (i.e., between 6.5 and 7.5);
3. Sensitive: APTI < mean APTI − SD (i.e., < 6.5).

To establish these categories, the mean APTI and SD were calculated separately for each tree species from multiple locations, ensuring species‐specific tolerance ranges.

### 2.7. Anticipated Performance Index of Studied Tree Species

For each tree species, an API was determined based on the APTI value and some important sociobiological characteristics [27, 48] (Table 2). This study adopted the API methodology as described [26, 45] (Table 3) and a score was assigned to each tree species. Similar approaches have been applied, for example, by [42, 49] in selecting tree species for greenbelt biomonitoring. According to this scoring system, each tree can receive a maximum of 16 points, and then expressed as a percentage as follows:

API Score Grades obtained by plant speciesMaximum possible grades of any species×100%

**Table 2:** Grading trees based on APTI, morphology, and socio‐economic significance.

**Grading character**	**Parameters**	**Pattern of assessment**	**Grade allotment (point)**
Tolerance	APTI	Less than 6.5	1
		Between 6.5 and 7.5	2
		Above 7.5	3 (maximum)

Morphological	Type of tree	Deciduous	0
		Semi‐deciduous	
		Evergreen	1 (Maximum)
	Tree size (height	Small: less than 30 ft (< 9.14 m)	0
		Medium: 30–70 ft (9.17–21.34 m)	1
		Large: above 70 ft (21.36 m)	2 (Maximum)
	Canopy structure	Sparse, irregular, globular	0
		Spreading crown, open, semi dense	1
		Spreading dense	2 (Maximum)
	Laminar structure		
	(a) Leaf size	Less than 225 mm^2^	1
		225–2025 mm^2^	2
		2025–4500 mm^2^	3
		4500–18225 mm^2^	4 (maximum)
	(b) Texture	Smooth	0
		Rough, coriaceous, leathery	1 (maximum)
	(c) Hardness	Soft, delicate, fragile	0
		Hardy, robust, sturdy	1 (maximum)

Socioeconomic value	Food, fodder, timber,	Less than three uses	0
	Medicinal, raw material, erosion control,	Three or more uses	1
	Shade/shelter, ornamental, Reclamation	Five or more uses	2 (maximum)

*Note:* Source: [26, 45, 49].

**Table 3:** Anticipated performance index (API) of tree species.

**Grade**	**Score (%)**	**Assessment category**
0	Up to 30	Not recommended
1	31–40	Very poor
2	41–50	Poor
3	51–60	Moderate
4	61–70	Good
5	71–80	Very good
6	81–90	Excellent
7	91–100	Best

*Note:* Source: [26, 45, 49].

This percentage is then used to determine the API value category, such as not recommended, very poor, poor, moderate, good, very good, excellent, and best [37] for that tree species when used as bioindicator of air pollution (Table 3).

### 2.8. Data Analysis

The Shapiro–Wilk and Levene tests were utilized to evaluate the normality and homogeneity of variance of quantitative data, respectively. One‐way analysis of variance (ANOVA) and mean comparisons using Tukey’s test at a significance threshold of *p* = 0.05 were used to identify statistical differences in the data. Pearson correlation was performed between APTI and physio‐biochemical parameters in which results were considered significant at 95% confidence interval. Principal component analysis (PCA) was used to identify which parameters most influence APTI.

## 3. Results

### 3.1. Air Quality of Sampling Roads

The mean air quality in the city of Winneba during the study period is presented in Table 4.

**Table 4:** Mean and SD air quality in Winneba during the study period.

**Sampling roads**	**CO(*μ*g/m** ^**3**^ **)**	**NO**_**x**_ **(*μ*g/m** ^**3**^ **)**	**PM**_**2.5**_ **(*μ*g/m** ^**3**^ **)**	**PM**_**10**_ **(*μ*g/m** ^**3**^ **)**
Highway I	2125 ± 182.40^a^	185 ± 95.73^a^	871 ± 79.54^a^	931 ± 51.29^a^
Highway II	1783 ± 780.25^a^	181 ± 87.88^a^	877 ± 82.53^a^	875 ± 53.79^a^
Highway III	1804 ± 792.43^a^	198 ± 27.01^a^	879 ± 81.80^a^	884 ± 15.04^a^
Control	821 ± 485.87^a^	168 ± 51.926^a^	902 ± 107.16^a^	874 ± 90.42^a^
EPA‐Ghana Reference/24 h	72000	150	35	70

*Note:* Mean ± SD, *n* = 3 in the same column with different letters in superscript are significantly different (*p* ≤ 0.05) [35].

### 3.2. RWC (%)

Variation in RWC across sampling locations is presented in Table 5. For *A. lebbeck*, RWC ranged significantly from 59.36% at Highway I to 83.22% at control road, with statistically significant differences indicated by the superscripts “a” and “b”. *A. indica* on the contrary, exhibited relatively stable RWC values across most locations, with no statistically significant differences (*p* > 0.05). The highest RWC (88.42*%* ± 1.37*%*) was recorded at Highway II, while the lowest (68.97*%* ± 8.51*%*) was observed at Highway I. *K. senegalensis* exhibited the lowest RWC among the studied species, with values ranging from 53.80*%* ± 19.89*%* at Highway I to 77.29*%* ± 13.82*%* at Highway II. *S. siamea* exhibited high RWC values across all locations, with the highest recorded at Highway II (89.36*%* ± 14.13*%*) and the lowest at Highway III (72.69*%* ± 36.33*%*). While no statistically significant differences were observed across the roads.

**Table 5:** Mean and SD of relative water content (%) of the tree species.

**Sampling road**	**Tree species**			
	***A. lebbeck***	***A. indica***	***K. senegalensis***	***S. siamea***
Highway I	59.36 ± 10.45^a^	68.97 ± 8.51^a^	53.80 ± 19.89^a^	78.29 ± 15.96^a^
Highway II	73.81 ± 2.06^ab^	88.42 ± 1.37^a^	77.29 ± 13.82^a^	89.36 ± 14.13^a^
Highway III	71.48 ± 5.70^ab^	70.21 ± 24.36^a^	N/P	72.69 ± 36.33^a^
Control road	83.22 ± 12.54^b^	68.703 ± 14.19^a^	60.97 ± 41.06^a^	83.33 ± 28.87^a^

*Note:* Mean ± SD, *n* = 3 in the same column with different letters in superscript are significantly different (*p* ≤ 0.05); N/P = Not present at location.

### 3.3. Leaf Extract pH

The pH variations of leaf extracts across tree species and sample roads are presented in Table 6. *A. lebbeck* exhibited significantly higher pH values at polluted roads (Highways I, II, and III) compared with the control road. The pH ranged from 6.40 ± 0.24 to 6.77 ± 0.14 at the highways, while it was significantly lower (5.13 ± 0.03) at the control road. The pH of *A. indica* leaves remained relatively stable across all locations, ranging from 6.36 ± 0.23 at Highway I to 6.55 ± 0.45 at the control road. *K. senegalensis* showed a notable increase in pH at the control road (6.63 ± 0.01), significantly higher than the values at Highways I and II (5.99 ± 0.22 and 6.12 ± 0.12, respectively). *S. siamea* exhibited the lowest pH values at highways, ranging from 4.48 ± 0.41 at Highway II to 4.99 ± 0.32 at Highway III. At the control road, the pH was significantly higher (6.05 ± 0.12), indicating reduced stress.

**Table 6:** Mean and SD of leaf extract pH of the selected tree species.

**Sampling road**	**Tree species**			
	***A. lebbeck***	***A. indica***	***K. senegalensis***	***S. siamea***
Highway I	6.69 ± 0.19^b^	6.36 ± 0.23^a^	5.99 ± 0.22^a^	4.97 ± 0.38^a^
Highway II	6.40 ± 0.24^b^	6.37 ± 0.04^a^	6.12 ± 0.12^a^	4.48 ± 0.41^a^
Highway III	6.77 ± 0.14^b^	6.49 ± 0.17^a^	N/P	4.99 ± 0.32^a^
Control road	5.13 ± 0.03^a^	6.55 ± 0.45^a^	6.63 ± 0.01^b^	6.05 ± 0.12^b^

*Note:* Mean ± SD, *n* = 3 in the same column with different letters in superscript are significantly different (*p* ≤ 0.05); N/P = Not present at location.

### 3.4. Plant Leaf Ascorbate Content

The mean AA contents (mg/g) of four tree species sampled along highways and a control road is presented in Table 7. *A. lebbeck* consistently recorded the highest AA content, with a peak value of 0.055 mg/g at Highway I. In contrast, *A. indica* displayed consistently low AA levels across all sampling locations, with a maximum value of 0.008 mg/g at Highway II. Across all species, the AA content was higher at polluted highway sites than at the control road, though the differences were not statistically significant (*p* ≤ 0.05). *S. siamea* exhibited moderate AA levels, peaking at 0.027 mg/g at Highways I and II, but with a marked decline at the control road (0.008 mg/g).

**Table 7:** Mean and SD of ascorbic acid contents (mg/g) of selected tree species.

**Sampling road**	**Tree species**			
	***A. lebbeck***	***A. indica***	***K. senegalensis***	***S. siamea***
Highway I	0.055 ± 0.04^a^	0.005 ± 0.00^a^	0.020 ± 0.02^a^	0.027 ± 0.02^a^
Highway II	0.018 ± 0.01^a^	0.008 ± 0.01^a^	0.007 ± 0.01^a^	0.027 ± 0.02^a^
Highway III	0.005 ± 0.00^a^	0.004 ± 0.00^a^	N/P	0.010 ± 0.00^a^
Control road	0.003 ± 0.00^a^	0.004 ± 0.00^a^	0.003 ± 0.00^a^	0.008 ± 0.01^a^

*Note:* Mean ± SD, *n* = 3 in the same column with different letters in superscript are significantly different (*p* ≤ 0.05); N/P = Not present at location.

### 3.5. TC Content of Selected Plant Species

The TC content of the selected tree species varied significantly across the sampling locations as presented in Table 8. The chlorophyll content of *A. lebbeck* ranged from 2.032 at Highway I to 25.246 mg/g at the control road. The highest chlorophyll content was recorded at the control road. The chlorophyll content of *A. indica* ranged from 3.141 at Highway II to 11.336 mg/g at the control road and remained relatively stable across roads, with no significant differences (*p* > 0.05). *K. senegalensis* exhibited consistently low chlorophyll levels, with values ranging from 1.538 at Highway I to 4.248 mg/g at the control road. *S. siamea* showed moderate chlorophyll levels, ranging from 4.592 at Highway II to 8.790 mg/g at the control road, with no statistically significant differences across locations (*p* > 0.05). In total, the control road consistently recorded the highest chlorophyll levels across all species.

**Table 8:** Mean and SD of total chlorophyll (mg/g) content of selected tree species.

**Sampling road**	**Tree species**			
	***A. lebbeck***	***A. indica***	***K. senegalensis***	***S. siamea***
Highway I	2.032 ± 1.98^a^	7.513 ± 3.18^a^	1.538 ± 0.26^a^	8.374 ± 6.81^a^
Highway II	7.919 ± 5.19^ab^	3.141 ± 2.45^a^	2.862 ± 2.76^a^	4.592 ± 2.54^a^
Highway III	5.735 ± 5.75^ab^	6.933 ± 0.68^a^	N/P	4.788 ± 2.36^a^
Control road	25.246 ± 15.40^b^	11.336 ± 4.79^a^	4.248 ± 4.61^a^	8.790 ± 3.95^a^

*Note:* Mean ± SD, *n* = 3 in the same column with different letters in superscript are significantly different (*p* ≤ 0.05); N/P = Not present at location.

### 3.6. APTI and Classification for Selected Tree Species

The APTI and the classification of selected tree species across the locations is presented in Table 9. The mean APTI values of the four tree species varied from 5.39 to 8.96. *S. siamea* had highest APTI values ranging from 7.28 to 8.96. The total mean APTI value of each tree species followed the order: *S. siamea* (8.11) > *A. indica* (7.42) > *A. lebbeck* (7.22) > *K. senegalensis* (6.41). According to the APTI index, the tree species were classified using the range (5.39–8.96) as: (i) tolerant (> 7.5), (ii) intermediate (6.5–7.5), and (iii) sensitive (APTI < 6.5). *A. indica* was only classified as tolerant at Highway II and intermediate at the other locations, whereas *S. siamea* consistently exhibited tolerance on all highways with the exception of Highway III. *K. senegalensis* was sensitive in other places, particularly at Highway I and the control road, but showed tolerance at Highway II. Varied responses were noted for *A. Lebbeck*; it was tolerant at the control road, sensitive at Highway I, and intermediate at Highways II and III. In general, *K. senegalensis* was the most sensitive species on the highways, while *S. siamea* was the most resilient.

**Table 9:** Air pollution tolerance index and classification for selected tree species.

**Tree species**	**Relative water content (%)**	**pH**	**Ascorbic acid (mg/g)**	**Total chlorophyll (mg/g)**	**APTI**	**Classification**
Highway I						
*A. lebbeck*	59.36	6.69	0.055	2.032	5.98	Sensitive
*A. indica*	68.97	6.36	0.005	7.513	6.90	Intermediate
*K. senegalensis*	53.80	5.99	0.020	1.538	5.39	Sensitive
*Senna siamea*	78.29	4.97	0.027	8.374	7.87	Tolerant
Highway II						
*A. lebbeck*	73.81	6.04	0.018	7.919	7.41	Intermediate
*A. indica*	88.42	6.37	0.008	3.141	8.85	Tolerant
*K. senegalensis*	77.29	6.12	0.007	2.862	7.74	Tolerant
*S. siamea*	89.36	4.48	0.027	4.592	8.96	Tolerant
Highway III						
*A. lebbeck*	71.48	6.77	0.005	5.735	7.15	Intermediate
*A. indica*	70.21	6.49	0.004	6.933	7.03	Intermediate
*S. siamea*	72.69	4.99	0.010	4.788	7.28	Intermediate
Control road						
*A. lebbeck*	83.22	5.13	0.003	25.246	8.33	Tolerant
*A. indica*	68.70	6.55	0.004	11.336	6.88	Intermediate
*K. senegalensis*	60.97	6.63	0.003	4.248	6.10	Sensitive
*S. siamea*	83.33	6.05	0.008	8.790	8.34	Tolerant

### 3.7. API of Tree Species Along the Highways

The gradation of the tree species based on their APTI, morphological, and socioeconomic traits is presented in Table 10. To improve the green belt, the tree species that fit the grading model in terms of their API were suggested. Using the score classification for the API provided in Table 2, the API scores ranged from 68.75% (*A. lebbeck* and *S. siamea*) to 81.25% (*A. indica*), categorizing the species as ranging from Good to Excellent in performance. *A. indica* had the highest API grade of 6 (Excellent) with API percentage of 81.25. Conversely, *K. senegalensis* received a “Very Good” rating (75%), while *A. lebbeck* and *S. siamea* were rated as “Good”.

**Table 10:** API of trees based on APTI, morphological traits, and socioeconomic value.

**Grading parameter**	***A. lebbeck***	***A. indica***	***K. senegalensis***	***S. E***
APTI	1	1	1	1
Type of plant	1	1	1	1
Plant size (height)	2	2	2	2
Canopy structure	1	2	0	1
Laminar structure				
(a) Leaf size	3	4	4	3
(b) Texture	0	0	1	0
(c) Hardness	1	1	1	1
Socioeconomic value	2	2	2	2
Total (+)	11	13	12	11
API (%)	68.75	81.25	75	68.75
API (grade)	4	6	5	4
Assessment	Good	Excellent	Very good	Good

### 3.8. Correlation Between Biochemical Parameters and APTI of the Tree Species

The linear regression between biochemical properties and APTI of *A. lebbeck*, *A. indica*, and *K. senegalensis* from various locations is presented Table 11. Water retention capacity is a critical ecological characteristic supporting pollution tolerance, as evidenced by the consistently strong and statistically significant positive correlations between RWC and APTI across all road types (r ≥ 0.999; *p* < 0.05). There was moderate to strong negative correlations (ranging from −0.477 to −0.814) found between leaf extract pH and APTI across all sites. Depending on site‐specific circumstances, AA, a crucial antioxidant molecule, showed varying correlations with APTI. The moderate correlation at the control road (r = 0.517) and the strong positive association at Highway III (r = 0.941) imply that AA accumulation might be a specific physiological reaction to oxidative stress brought on by high vehicle emissions.

**Table 11:** Correlation between the APTI values and physio‐biochemical parameters.

**Biochemical parameter**	**Highway I**	**Highway II**	**Highway III**	**Control road**
	**APTI**	**APTI**	**APTI**	**APTI**
RWC	1.000 ^∗∗^	1.000 ^∗∗^	0.999 ^∗^	1.000 ^∗∗^
PH	−0.665	−0.477	−0.798	−0.814
AA	−0.228	0.267	0.941	0.517
TC	0.947	−0.512	−0.996	0.653

∗∗Correlation is significant at the 0.01 level (2‐tailed).

∗Correlation is significant at the 0.05 level (2‐tailed).

The relationships between TC and APTI also varied by site. Strong negative correlations were found at Highway II (r = –0.512) and Highway III (r = –0.996), whereas strong positive correlations were found at Highway I (r = 0.947) and the control site (r = 0.653). These results show that the synthesis or degradation of chlorophyll is extremely sensitive to pollution stress. Chlorophyll breakdown may be accelerated by high pollution levels (such as Highway III), which would lower photosynthetic capacity and negatively impact plant performance. On the other hand, species may preserve or even increase the amount of chlorophyll in less polluted areas, promoting higher APTI values and ecological function. This variation implies that, depending on the degree of exposure, chlorophyll levels may function as a bioindicator of pollution stress as well as a stand‐in for plant vitality.

### 3.9. Principal Component Analysis (PCA) of the Influence of Biochemical Parameters on APTI/API

The influence of biochemical parameters on APTI/API is presented in Table 12. The PCA communalities indicated that AA (99.6%), APTI (97.8%), RWC (97.7%), and TC (94.9%) were strongly represented by the extracted components, highlighting their importance in explaining species’ responses to pollution. In contrast, leaf pH (59.2%) showed a weaker representation, suggesting limited discriminatory power compared with other parameters at Highway I.

**Table 12:** Principal component analysis to identify most influencing parameters.

**Variable**	**Highway I**			**Highway II**			**Highway III**			**Control**		
	**Comm. (%)**	**PCA 1**	**PCA 2**	**Comm. (%)**	**PCA 1**	**PCA 2**	**Comm. (%)**	**PCA 1**	**PCA 2**	**Comm. (%)**	**PCA 1**	**PCA 2**
RWC	97.7	0.970	0.192	95.7	0.917	−0.342	100	1.00	0.014	99.6	0.972	0.225
TC	94.9	0.968	−0.106	85.3	0.334	0.861	100	−0.998	−0.064	99.1	0.359	0.932
APTI	97.8	0.966	0.211	95.6	0.921	−0.330	100	1.00	−0.024	99.9	0.972	0.224
PH	59.2	−0.756	−0.142	91.1	−0.782	−0.547	100	−0.783	0.622	96.0	−0.925	0.323
AA	99.6	−0.441	0.896	99.9	0.592	0.805	100	0.866	0.500	99.8	0.801	−0.591

At Highway II, AA (99.9%) and pH (91.1%) emerged as the most influential variables, while RWC (95.7%) and APTI (95.6%) also contributed substantially. TC, however, explained a relatively lower proportion of variance (85.3%), implying reduced sensitivity compared with other parameters. All variables were fully represented (100% communality) at Highway III, indicating that the biochemical traits jointly captured the variation in APTI/API. This suggests that under severe pollution conditions, each parameter, including pH and AA, becomes important in explaining species’ tolerance and response mechanisms.

At the control road, AA (99.8%), APTI (99.9%), RWC (99.6%), and TC (99.1%) again exhibited very high communalities, reaffirming their dominant role in explaining variance under less polluted conditions. Leaf pH also performed strongly (96.0%), indicating that in cleaner environments it aligns more closely with other biochemical markers in explaining species performance. Overall, PCA revealed that AA, RWC, APTI, and TC are consistent drivers of variation across sites, whereas leaf pH showed site‐dependent importance—weak at heavily polluted Highway I but much stronger under less polluted conditions.

## 4. Discussion

### 4.1. Species‐Level Variation in Biochemical Parameters

There was considerable variability in the RWC responses of plants across all locations, underscoring the site‐specific nature of plant physiological adaptations. A relatively stable RWC recorded in *A. indica*. In the literature, plant species capable of maintaining physiological balance under stress conditions, such as drought and air pollution, can retain internal water reserves [50]. *S. siamea* exhibited the highest RWC values across all locations, while *K. senegalensis* recorded the lowest (Table 5). High RWC has been shown to contribute to both drought and pollution resistance [12, 27, 36]. As a result, a RWC provides an adaptive advantage against environmental stress [9, 11, 51]. A high average RWC percentage not only helps dilute pollution‐induced acidity [52, 53], but it is also directly linked to protoplasmic permeability. Mei et al. [54] further suggested that RWC loss in leaves is associated with the accumulation of PM on the leaf surface.

In this study, *A. lebbeck* demonstrated significantly higher pH values, ranging from 6.40 to 6.77 at highways, compared with the reference road. Literature suggests that optimal leaf pH is essential for regulating internal biochemical functions [9, 55]. Again, high pH may improve the efficiency of conversion from hexose sugar to AA [36, 55, 56]. Nonetheless, plants with a near‐neutral pH (around 7) tend to exhibit higher pollution tolerance [11]. This suggests that *A. lebbeck* at Highway III with pH 6.77 may be more tolerant to air pollution than those at other highway locations (Table 4). Conversely, *S. siamea* exhibited lower pH (4.48–4.99) values across all highways (I to III), indicating higher susceptibility to pollution. This outcome concurs with previous studies that have shown that plant extracts from roadside environments tend to exhibit lower (more acidic) pH values because of air pollution [57].

*A. lebbeck* consistently recorded the highest AA content, particularly at Highway I (Table 5). The elevated AA levels observed in *A. lebbeck* may be attributed to the plant’s defense response against oxidative stress [14]. Conversely, *A. indica* exhibited consistently low AA levels, while *S. siamea* maintained moderate levels across all sampling locations. Plants with lower AA content may be at a greater risk of air pollution damage, as suggested by [58]. Across all studied tree species, AA content was generally higher at polluted highways than at the control road. Similar findings were reported by Banerjee et al. [9], who observed that *Ficus religiosa* and *Lagerstroemia speciosa* exhibited increased AA levels ranging 6.08–82.80 mg/g and 6.04–230.37 mg/g, respectively, in polluted environments, suggesting an adaptive response to air pollution stress. Uka et al. [49] have reported similar findings of increased AA content ranged between 10.91 and 19.81 mg/g.

Chlorophyll is essential for photosynthetic activity, its reduction has been widely used as an indicator of air pollution [59–61]. In this study, we observed no significant variation in TC content among the same tree species across different sampling roads. However, chlorophyll levels declined along highways compared with the control road.

This aligns with the findings of Joshi and Swami [59], who reported a reduction in chlorophyll a, b, and carotenoid content in leaf samples collected from polluted areas compared with those from control sites. According to Verma et al. [62], the biochemical, morphological, and physiological characteristics of plants may be impacted by changes in TC content. Typically, elevated concentrations of vehicular emissions, particularly SO₂ and NO_x_, degrade chlorophyll molecules, reducing photosynthetic efficiency and affecting biochemical responses [9, 11, 63] also demonstrated that exposure to SO₂ and O₃ lowered chlorophyll concentrations in various crop plants. Similarly, studies [64–66] linked acidic pollutants such as SO₂ to chlorophyll degradation, leading to the formation of phaeophytin through chlorophyll acidification.

### 4.2. Role of APTI and API in Air Pollution Mitigation

APTI determines whether a plant species is tolerant or sensitive to air pollution, with higher values indicating greater tolerance and lower values signifying sensitivity [11, 59]. In this study the tree species were classified as tolerant (> 7.5), intermediate (6.5–7.5), and sensitive (APTI < 6.5). Accordingly, *S. siamea* and *A. indica* were categorized as tolerant, *A. lebbeck* as intermediate, and *K. senegalensis* as sensitive. This finding aligns with the hypothesis that plant responses to air pollution vary by species and location [67]. Regional studies provide further insight into species‐specific responses. For instance, Acebron et al. [68] reported that *A. indica* exhibited greater tolerance to roadside air pollution in Jülich, Germany. Similarly, Shahid et al. [29] found *A. indica* to be pollution‐tolerant in Karachi, Pakistan, while confirming *A. lebbeck* as pollution‐sensitive, contrary to the findings of this study. Consistent with our findings, a study by Hassan et al. [30] in Zaria‐Kaduna, Nigeria, identified *S. siamea* as pollution‐tolerant and *A. indica* as sensitive, highlighting geographical variability in plant responses. *S. siamea* consistently recorded the highest APTI values ranging between 7.28 and 8.96 (Table 7) among the studied species indicating relatively better adaptation to polluted environments. Our study presents the first account of *K. senegalensis* as being sensitive to air pollution in this region, while little to no information is available on its pollution tolerance.

Using the API score classification in this study (Table 2), the tree species were categorized ranging from 68.75% to 81.25% (Good to Excellent) in performance. *A. indica* obtained the highest API score of 81.25%, earning an “Excellent” rating (Grade 6). According to Linden et al. and Yadav et al. [19, 69], for urban greening, trees with high API scores are often recommended because of their tolerance to air pollution. The high API score of *A. indica* reinforces previous findings that identify it as a resilient species capable of thriving in urban environments [24, 26]. Conversely, *K. senegalensis* received a “Very Good” rating while *A. lebbeck* and *S. siamea* were categorized as “Good” [70].

### 4.3. Cause–Effect Analysis Between APTI and Biochemical Parameters

APTI is influenced by variations in biochemical parameters, with correlations among RWC, AA, TC, and leaf extract pH playing a crucial role in determining pollution tolerance [11, 26]. Several studies have reported positive correlations between APTI and biochemical parameters, highlighting their role in assessing plant responses to air pollution [24, 51, 57]. Our earlier findings indicate a strong positive correlation was observed between RWC and APTI across all locations (Table 9) [70]. RWC is widely recognized as a key determinant of APTI [24, 45]. Conversely, APTI and leaf extract pH showed a negative correlation across all locations, though the relationship was not statistically significant (Table 9) [70]. This trend indicates that as leaf extract pH decreases, APTI also tends to decline, suggesting that leaf pH alone may not be a reliable predictor of pollution tolerance. Previous research has shown that tree species with lower leaf pH values are generally more sensitive to air pollution, which aligns with findings that vehicular emissions, particularly SO₂ and NO_x_, contribute to leaf acidification and biochemical stress [46, 71].

The relationships between AA, TC, and APTI varied across the studied roads, with some correlations being strongly positive and others strongly negative, though none reached statistical significance (Table 9) [70]. Similar variability in AA content across species and environmental conditions has been reported by Ogunkule et al. [42], emphasizing the need for site‐specific assessments of APTI. AA increase has been shown to significantly influence APTI [13, 47]. A similar trend was observed in the relationship between APTI and TC in our earlier report [70]. Giri et al. [72] noted that chlorophyll levels fluctuate over time because of variations in weather conditions and pollution stress, which is consistent with the findings of Nkansah et al. [70], who reported fluctuating levels of vehicular pollutants such as CO, NO_x_, PM₂.₅, and PM₁₀ in the study region. These variations further support the assertion that environmental conditions play a critical role in shaping the biochemical responses of trees to pollution. The findings of this study align with Ghana’s Environmental Protection Agency (EPA) objectives on urban air quality management and the Forestry Commission Green Ghana Project, which seeks to promote urban tree planting as part of national climate resilience and pollution abatement strategies [73].

## 5. Conclusion and Future Perspective

This study assessed the physiological, biochemical, and tolerance responses of four commonly occurring tree species (*A. lebbeck*, *A. indica*, *K. senegalensis*, and *S. siamea*) for their potential role in air pollution mitigation and biomonitoring in Winneba. The results indicated that the tree species responded differently to pollution stress, with *S. siamea* and *A. indica* showing greater resilience, while *K. senegalensis* and *A. lebbeck* were more sensitive under urban highway vehicular pollution. Variation in RWC, leaf pH, AA concentration, and chlorophyll levels influenced the APTI and API, which provided the basis for the trees’ classification. Overall, *A. indica* emerged as a suitable candidate that can be integrated into roadside and median plantings, green belts, and vegetative buffers [74, 75]. It had stable biochemical traits and high API ranking, while *S. siamea* showed promise as a bioindicator species. Conversely, the sensitivity of *A. lebbeck* and *K. senegalensis* highlights their usefulness in monitoring pollution‐induced stress rather than pollution mitigation. This study contributes to Ghana’s progress toward the United Nations Sustainable Development Goals, particularly SDG 11 (Sustainable Cities and Communities) and SDG 13 (Climate Action), by demonstrating how pollution‐tolerant urban trees can enhance green infrastructure and mitigate climate‐related air quality challenges. Future work should incorporate long‐term monitoring of seasonal changes, particulate matter deposition studies, and advanced approaches such as remote sensing and machine learning for large‐scale species classification. Such integrated strategies will strengthen the role of urban trees as both active mitigators of pollution and reliable bioindicators for sustainable urban ecosystem management.

### 5.1. Takeaway Message

The selection of tree species for air pollution mitigation should be based on a multicriteria approach integrating physiological tolerance, bioindication potential, and socioeconomic value to maximize environmental benefits.

## Ethics Statement

This study did not involve humans or animals as subjects; there was no harm anticipated to human or animal life. Ethical issues (Including plagiarism, misconduct, data fabrication and/or falsification, double publication and/or submission, redundancy, etc.) have been completely observed by the authors.

## Disclosure

All authors read and approved the final manuscript.

## Conflicts of Interest

The authors declare no conflicts of interest.

## Author Contributions

Conceptualization: F.K.N., E.J.D.B., and J.N.H.; Methodology: F.K.N., E.J.D.B., and J.N.H., Formal analysis: F.K.N.; Investigation: F.K.N.; Resources: F.K.N.; Data curation: F.K.N., E.J.D.B., and J.N.H.; Writing – original draft preparation: F.K.N.; Writing – review and editing: E.J.D.B., and J.N.H.; Supervision: E.J.D.B. and J.N.H.

## Funding

No funding was received for this manuscript.

## Data Availability

Data supporting the results of this study are available from the corresponding author upon request.
